# Efficacy of mobile apps for urinary incontinence in women: a meta-analysis of randomized controlled trials

**DOI:** 10.3389/fpubh.2026.1859960

**Published:** 2026-08-18

**Authors:** Wen Zhao, Xiuxiu Cui, Lu Bai

**Affiliations:** 1 Department of Pelvic Floor Medicine Center Nursing, West China Second University Hospital, Sichuan University, Chengdu, China; 2 Key Laboratory of Birth Defects and Related Diseases of Women and Children (Sichuan University), Ministry of Education, Chengdu, Sichuan, China; 3 Department of Outpatient Treatment Nursing, West China Second University Hospital, Sichuan University, Chengdu, China

**Keywords:** adherence, meta-analysis, mobile apps, pelvic floor muscle training (PFMT), urinary incontinence

## Abstract

**Objective:**

Pelvic floor muscle training (PFMT) is recommended as a first-line treatment for urinary incontinence (UI); however, treatment adherence among women with UI remains suboptimal. The effectiveness of mobile applications in delivering PFMT for women with UI is not yet well established. This study aimed to evaluate the effects of app-based PFMT interventions on adherence, symptom severity, quality of life, and patient global impression of improvement (PGI-I).

**Methods:**

PubMed, Cochrane Library, Scopus, EMBASE, and Web of Science were systematically searched to identify relevant studies published up to February 11, 2026. Risk ratios (RRs) and mean differences (MDs) with 95% confidence intervals (CIs) were calculated.

**Results:**

A total of 12 randomized controlled trials (RCTs) including 1,647 participants (mobile app group: 823; usual care group: 824) were included. The meta-analysis demonstrated that, compared with usual care, mobile app–based interventions significantly reduced the severity of UI (MD = −1.93, 95% CI: −2.87, −0.98; *P* < 0.0001) and the frequency of incontinence episodes (MD = −2.28, 95% CI: −3.62, −0.94; *P* = 0.0008). In addition, these interventions improved adherence (RR = 1.42, 95% CI: 1.26, 1.59; *P* < 0.00001), PGI-I (RR = 3.79, 95% CI: 1.27, 11.29; *P* = 0.02), and quality of life (MD = −2.78, 95% CI: −4.43, −1.14; *P* = 0.0009).

**Conclusions:**

This meta-analysis suggests that mobile app-based PFMT interventions may serve as an adjunctive approach to improve adherence and patient-reported outcomes among women with UI. However, the magnitude of symptom improvement may be below the threshold of clinical importance, and the certainty of evidence remains low. Mobile applications should therefore complement rather than replace supervised PFMT, particularly for women requiring individualized rehabilitation and objective functional assessment.

**Systematic review registration:** https://www.crd.york.ac.uk/PROSPERO/view/CRD420261472595, identifier CRD420261472595.

## 1 Introduction

The International Continence Society (ICS) defines urinary incontinence (UI) as any involuntary leakage of urine (1). Epidemiological data indicate that the prevalence of UI among adult women worldwide ranges from 25 to 45%, with rates as high as 45.1% reported in Asia (2, 3). UI is commonly associated with multiple risk factors, including smoking, ethnicity, menopause, obesity, pregnancy, childbirth-related pelvic floor dysfunction, hysterectomy, chronic cough, history of miscarriage, and prior surgical procedures (3, 4). UI imposes a substantial burden on women, adversely affecting physical health, psychological wellbeing, sexual function, work productivity, and overall quality of life. Moreover, it increases the risk of urinary tract infections and contributes to rising healthcare costs (3–5). In the United States, the annual medical expenditure attributable to UI is estimated to exceed $900 per patient (5).

Current management strategies for UI primarily include pelvic floor muscle training (PFMT), bladder training, and lifestyle modifications (5, 6). Evidence suggests that supervised PFMT can achieve satisfactory symptom improvement (7, 8). However, adherence remains a critical determinant of treatment success, as sustained and correct implementation of PFMT is required to achieve optimal outcomes (4). In practice, supervised PFMT often necessitates frequent clinical visits, which are time-consuming and costly, thereby limiting long-term adherence (8). Indeed, adherence to PFMT remains suboptimal, with only approximately 49% of women with UI performing standardized and correct exercises, and merely 23% maintaining long-term adherence (3).

In recent years, with the rapid advancement of information technology, mobile health (mHealth) interventions have demonstrated increasing potential for improving the management of various chronic conditions (9, 10). mHealth not only delivers disease-related education and serves as an adjunct to conventional therapy but also enables access to interventions without temporal or geographical constraints (5). In particular, mobile apps offer advantages such as ease of use, cost-effectiveness, convenience, and the ability to provide personalized exercise programs and reminders (11). Consequently, mobile apps are considered a promising tool to enhance self-management among women with UI. However, current evidence regarding the effectiveness of mobile app interventions in improving UI outcomes remains inconsistent. Some randomized controlled trials (RCTs) have reported that mobile app-based interventions significantly improve UI symptoms and quality of life compared with usual care (6, 12, 13). In contrast, studies such as that by Loohuis et al. (14) found no significant improvements in symptom severity or quality of life with mobile app interventions.

Previous systematic reviews (3, 5) have evaluated telemedicine or broader digital health interventions for women with UI. However, these reviews combined heterogeneous intervention modalities, such as telephone-based support, web-based programs, and mobile applications, making it difficult to determine the specific effectiveness of mobile app-based PFMT. In addition, some previous reviews (8, 11) focused primarily on qualitative evidence synthesis without performing a quantitative meta-analysis, limiting the precision of the available evidence. Furthermore, several recently published RCTs were not included in earlier reviews. Therefore, we conducted an updated systematic review and meta-analysis focusing specifically on mobile app-based PFMT interventions in women with UI. In addition to evaluating UI symptom severity, adherence, quality of life, and patient global impression of improvement (PGI-I), we also synthesized evidence regarding pelvic floor muscle function and explored potential sources of heterogeneity through subgroup analyses. Our findings provide more focused and up-to-date evidence regarding the effectiveness of mobile app-based PFMT to support clinical decision-making and future app development.

## 2 Methods

### 2.1 Search strategy

This meta-analysis was conducted in accordance with the Preferred Reporting Items for Systematic Reviews and Meta-Analyses (PRISMA) guidelines. This meta-analysis was conducted in accordance with the Preferred Reporting Items for Systematic Reviews and Meta-Analyses (PRISMA) guidelines.

PubMed, Cochrane Library, Scopus, EMBASE, and Web of Science were systematically searched from inception to February 11, 2026. Search terms are presented in Table 1. Two authors (W.Z. and X.C.) independently performed the literature search. In addition, we checked the reference lists of the identified articles and related reviews to further screen for eligible studies. No time or language restrictions were applied during the search process.

**Table 1:** Electronic search strategy.

Database	Search strategy (published up to February 11, 2026)	Number
PubMed (all fields)	(stress urinary incontinence OR urinary incontinence) AND (Mobile applications OR Smart phone OR Smartphone OR Smartphone education OR Smartphone application OR cell phone OR phone) AND (randomized controlled trial OR RCT OR random OR randomized controlled trial OR Clinical Trial)	170
Embase (all fields)	(stress urinary incontinence OR urinary incontinence) AND (Mobile applications OR Smart phone OR Smartphone OR Smartphone education OR Smartphone application OR cell phone OR phone) AND (randomized controlled trial OR RCT OR random OR randomized controlled trial OR Clinical Trial)	185
Cochrane library trials (all fields)	(stress urinary incontinence OR urinary incontinence) AND (Mobile applications OR Smart phone OR Smartphone OR Smartphone education OR Smartphone application OR cell phone OR phone) AND (randomized controlled trial OR RCT OR random OR randomized controlled trial OR Clinical Trial)	131
Web of science (topic)	[TS = (stress urinary incontinence OR urinary incontinence)] AND [TS = (Mobile applications OR Smart phone OR Smartphone OR Smartphone education OR Smartphone application OR cell phone OR phone)] AND [TS = (randomized controlled trial OR RCT OR random OR randomized controlled trial OR Clinical Trial)]	145
Scopus (all fields)	(stress urinary incontinence OR urinary incontinence) AND (Mobile applications OR Smart phone OR Smartphone OR Smartphone education OR Smartphone application OR cell phone OR phone) AND (randomized controlled trial OR RCT OR random OR randomized controlled trial OR Clinical Trial)	152

### 2.2 Study selection

Inclusion criteria were as follows:

- Population: adult women (≥ 18 years) with the diagnosis of UI;
- Intervention: PFMT using mobile phone applications;
- Comparison: comparison group receiving usual care (e.g., conventional home-based training without mobile phone applications);
- Outcomes: the primary outcomes included UI severity, assessed using the International Consultation on Incontinence Questionnaire-Urinary Incontinence Short Form (ICIQ-UI SF); adherence; and quality of life, evaluated using the International Consultation on Incontinence Questionnaire-Lower Urinary Tract Symptoms Quality of Life (ICIQ-LUTSqol). Secondary outcomes included pelvic floor muscle function, incontinence episode frequency, and PGI-I;
- Study design: RCTs.

Reviews, laboratory studies, duplicate publications, letters, case reports, and conference abstracts were excluded.

### 2.3 Data extraction

Data from each eligible study were independently extracted by two authors (W.Z. and X.C.) using a standardized data collection form, and any discrepancies were resolved through consultation with a third author (L.B.). Extracted data included author name, year of publication, country, study design, study population (sample size and age), and outcomes (UI severity, adherence, quality of life, pelvic floor muscle function, incontinence episode frequency, and PGI-I).

### 2.4 Quality assessment

The risk of bias of the included RCTs was independently assessed by two reviewers (W.Z. and X.C.) using the Cochrane risk-of-bias tool 2: (1) randomization process, (2) deviations from intended interventions, (3) missing outcome data, (4) measurement of the outcome, (5) selection of the reported results, and (6) overall risk of bias. Any disagreements were resolved through discussion, with arbitration by a third author when necessary (L.B.). The evidence was graded using the Grading of Recommendations Assessment, Development and Evaluation (GRADE) framework.

### 2.5 Statistical analysis

Review Manager (RevMan, version 5.3) was used to perform this systematic review and meta-analysis. For continuous outcomes assessed using identical measurement scales across studies, mean differences (MDs) with corresponding 95% confidence intervals (CIs) were calculated, whereas risk ratios (RRs) with 95% CIs were used for dichotomous outcomes. Mean differences (MDs) were used because each pooled analysis included only studies that employed the same validated outcome measure (e.g., ICIQ-UI SF or ICIQ-LUTSqol), thereby preserving the clinical interpretability of absolute score differences. Statistical heterogeneity was assessed using the *I*^2^ statistic. An *I*^2^ value >50% was considered indicative of substantial heterogeneity, in which case a random-effects model was applied; otherwise, a fixed-effects model was used (15). Subgroup analyses were performed, when appropriate, according to app characteristics and intervention duration. Sensitivity analysis was conducted using a leave-one-out approach to evaluate the robustness of the pooled results. Publication bias was assessed by visual inspection of funnel plots and quantitatively using Egger's test. A *P* value < 0.05 was considered statistically significant.

## 3 Results

### 3.1 Study selection

**Figure 1:**
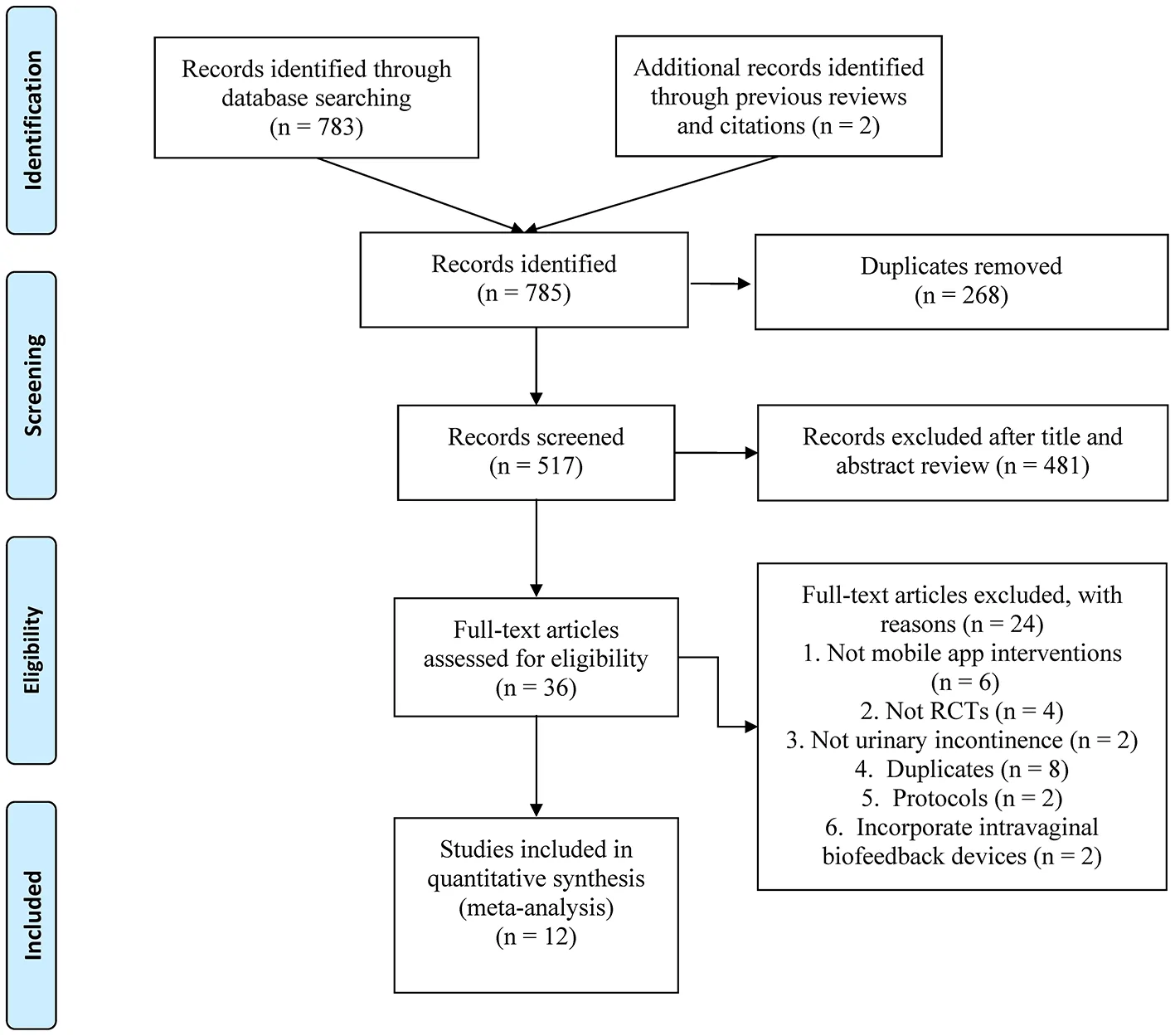
The PRISMA flowchart.

A total of 785 studies were identified from five databases, of which 268 duplicates were removed. Following title and abstract screening, 481 studies were excluded, and the full texts of the remaining 36 studies were assessed for eligibility. Ultimately, 12 studies (6, 12–14, 16–23) met the inclusion criteria and were included in the final analysis (Figure 1).

### 3.2 Study characteristics and quality assessment

**Figure 2:**
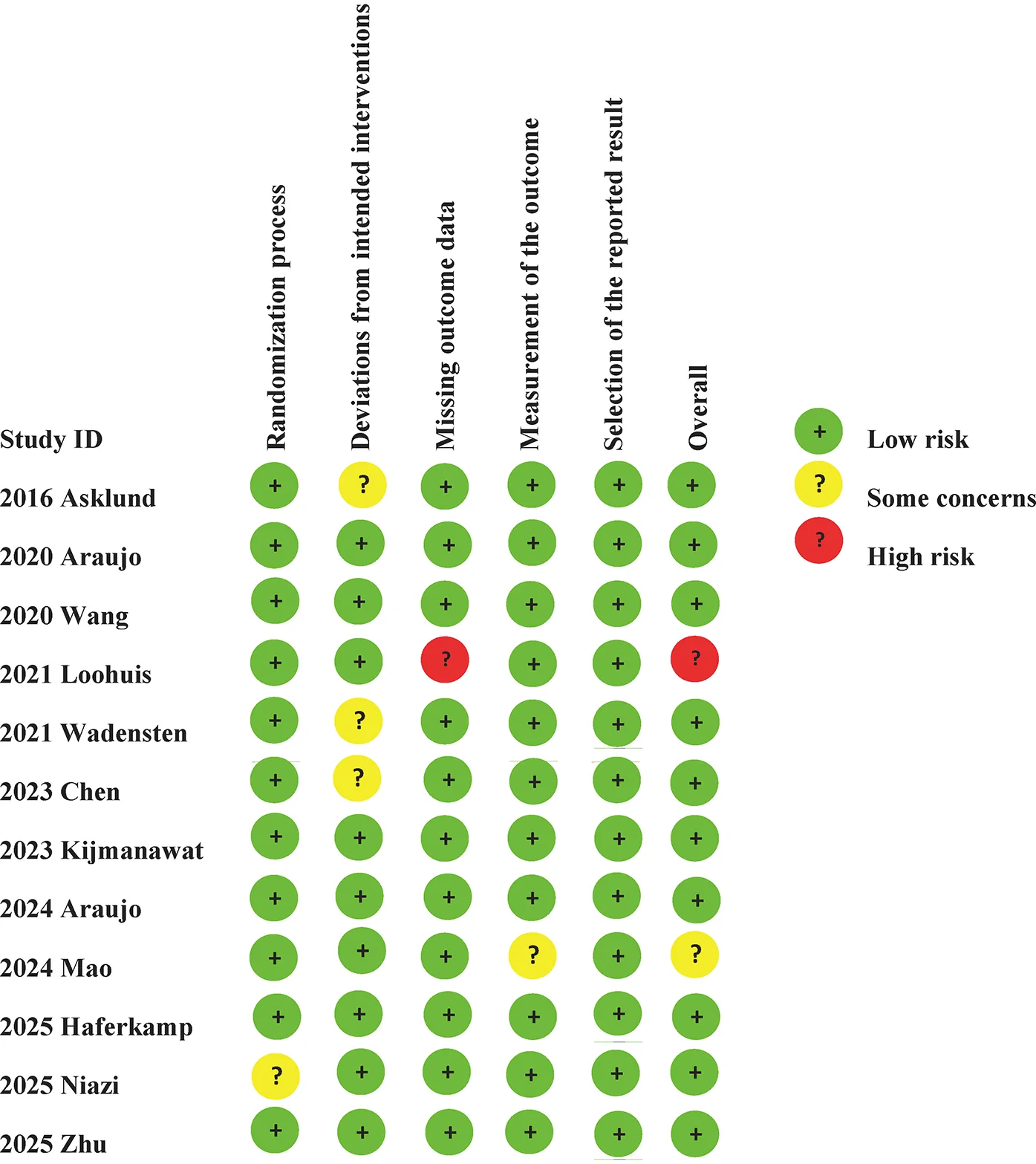
Risk of bias for each included study.

The main characteristics of the included studies are summarized in Table 2. The studies were published between 2016 and 2025, comprising a total of 1,647 participants (mobile app group: 823; usual care group: 824). These studies were conducted in multiple countries, including Sweden, the Netherlands, Brazil, China, Thailand, Germany, and Pakistan. Most included studies were judged to have a low overall risk of bias, whereas several studies raised some concerns in specific domains. The detailed risk-of-bias assessments are presented in Figure 2. In the GRADE assessments (Table 3), the certainty of evidence in the reported outcomes mostly ranged from low to high, due to concerns regarding statistical heterogeneity (inconsistency).

**Table 2:** Study characteristics of the 12 included studies.

References	Country	Sample size	Age	Type of UI	App name	App function	Comparative	Duration	Outcome measures
Asklund et al. (16)	Sweden	AG: 62 UG: 61	AG: 44.8 (9.7) UG: 44.7 (9.1)	SUI	Tät^®^	The app focused on PFMT exercises but also contained information that described SUI, the pelvic floor, and lifestyle factors related to incontinence	Usual care	3 months	ICIQ-UI SF; adherence; QoL; PGI-I; IEF
Araujo et al. (17)	Brazil	AG: 17 UG: 16	AG: 47.2 (10.6) UG: 53.3 (13.2)	SUI	Diário Saúde	The app was based on the visual component of sEMG as a guide for PFMT, without a vaginal probe but with better screen resolution and an alarm that reminds the used perform the exercises twice a day. At home, the women were asked to repeat the exercises by following a dynamic sequence of images on the app screen these images presented a correlation with the exercise that was being requested	Usual care	3 months	ICIQ-UI SF; PERFECT
Wang et al. (18)	China	AG: 54 UG: 54	AG: 29.2 (2.6) UG: 29.1 (2.9)	SUI	Pen Yi Kang	This app contains a home training module, a systematic audio guidance pelvic floor muscle training program, which was used for intervention. This training was ranked by increasing intensity with four levels (one primary training, two stage medium training, two stage advanced training and one mixed muscle training)	Usual care	3 months	ICIQ-UI SF
Loohuis et al. (14)	The Netherlands	AG: 131 UG: 131	AG: 53.2 (12.8) UG: 51.3 (10.2)	SUI, UUI or, MUI	URinControl	Contained a step-by-step program for the self-management of UI that was based on relevant Dutch general practitioner and international guidance for treating UI	Usual care	3 months	ICIQ-UI SF; QoL; PGI-I; IEF
Wadensten et al. (19)	Sweden	AG: 60 UG: 63	AG: 58.9 (9.2) UG: 57.7 (9.9)	UUI or, MUI	Tät II	The Tät II app is focused on four themes: PFMT, bladder training, psychoeducation, and lifestyle advice	Usual care	15 weeks	ICIQ-UI SF; QoL; PGI-I; IEF
Chen et al. (20)	China	AG: 63 UG: 63	AG: 28.35 (3.01) UG: 29.16 (3.01)	UI	UIW app	The app focuses on the PFMT module, where a staged training program is designed in the order of difficulty. The app also contains other modules, including Risk Assessment, Health Education, and an Online Evaluation forum, with the functions of education, reminders, consultation, self-monitoring, and others	Usual care	2 months	ICIQ-UI SF; QoL; IEF
Kijmanawat et al. (21)	Thailand	AG: 26 UG: 25	AG: 53.0 (9.3) UG: 56.5 (8.9)	SUI	-	PFMT with animations, recording system, and reminder system	Usual care	3 months	ICIQ-UI SF; adherence; IEF
Araujo et al. (26)	Brazil	AG: 76 UG: 78	AG: 61 (6.1) UG: 60.6 (6.8)	SUI or MUI	Diário Saúde^®^	The Diário Saúde^®^ app was designed for conducting home-based exercises and included visual electromyography components to guide PFMT. The app was developed using the visual component of surface electromyography as a guiding tool for PFMT	Usual care	4 months	ICIQ-UI SF; PERFECT
Mao et al. (13)	China	AG: 81 UG: 77	AG: 63.46 (6.70) UG: 63.49 (6.71)	SUI	-	Patients could obtain knowledge of pelvic floor muscles at any time through the app master lecture hall, and the information was presented in the form of text, pictures and videos; Home training was provided to: (a) deliver exercise reminders every day; (b) allow patients to choose from four levels of pelvic floor muscle rehabilitation training courses with increasing difficulty according to their own needs, and carry out pelvic floor muscle contraction and relaxation exercises through animated voice guidance; (c) automatically generate exercise data, including the time of each contraction and relaxation and total duration; (d) prompt the patient to adjust the irregular contraction and relaxation of muscle movements after connecting the vaginal dumbbells to ensure the optimal effect.	Usual care	3 months	Adherence
Haferkamp et al. (6)	Germany	AG: 96 UG: 98	AG: 49 (40-56) UG: 51 (39-57)	SUI, UUI or, MUI	Kranus Mictera	First, the app provided a bladder diary, through which participants could log fluid intake, meals, voiding volume and frequency, pad use, and urinary symptoms. An algorithm provided personalized suggestions and a visual representation that helped participants recognize patterns and build motivation. Second, the app provided pelvic floor and physiotherapy exercises. Third, the app provided bladder training and behavioral therapy through weekly content based on cognitive–behavioral principles, aiming to increase bladder capacity, reduce urgency, and regulate voiding frequency. Fourth, the app provided mental exercises, which involved weekly audio-guided sessions on mindfulness, progressive muscle relaxation, and stimulus controlto address stress and reflex inhibition. Fifth, the app provided acute urge control, a so-called urge-stop function that provided immediate access to short exercises and distraction games to manage acute urgency episodes. Sixth, the app provided daily educational content that covered bladder dysfunction, therapeutic strategies, and supportive diet and lifestyle modifications. Motivational strategies in the app were based on established behavioral change principles, including immediate positive feedback, goal-based badge rewards, daily streak tracking to support consistency and self-efficacy, and optional push notifications to prompt adherence. Finally, the app included progress tracking and reporting, which included adherence badges and a downloadable therapy report for use in clinical consultations	Usual care	3 months	ICIQ-UI SF; PGI-I; IEF
Niazi et al. (22)	Pakistan	AG: 10 UG: 10	AG: 36.4 (9.4) UG: 47.2 (8.8)	UI	Pelvi-Fit app	Pelvic Floor Muscle (PFM) exercises incorporated the Knack Maneuver and followed a structured protocol. The Pelvi-Fit app also included a patient education section, in which instructions were provided regarding the fluid intake, bladder irritants, and general lifestyle modifications, which were accessible throughout the training period	Usual care	6 weeks	ICIQ-UI SF
Zhu et al. (23)	China	AG: 147 UG: 148	AG: 29.05 (5.08) UG: 28.97 (5.10)	UI	UIW	(1) Risk assessment module: Using our postpartum incontinence prediction tool, this module assesses the risk of postpartum incontinence based on pregnant women's clinical characteristics. It provides feedback on individual risk levels to help identify high-risk groups. (2) Health knowledge module: This module covers topics such as UI, weight management, pregnancyrelated constipation, pelvic floor muscle health, and general pregnancy care. Information is delivered via papers, images, and videos. (3) Pelvic floor muscle training module: This module includes a 2-stage beta version as well as a comprehensive 4-stage PFMT program. Full details of the specific PFMT program are available in our previously published report. (4) Online assessment module: This module uses a validated scale to evaluate UI symptoms, quality of life, and self-efficacy in PFMT	Usual care	2 months	ICIQ-UI SF

AG, app group; UG, usual care group; ICIQ-UI SF, international consultation on incontinence questionnaire-urinary incontinence short form; ICIQ-LUTSqol, international consultation on incontinence questionnaire-lower urinary tract symptoms quality of life; MUI, mixed urinary incontinence; PFMT, pelvic floor muscle training; PGI-I, patient global impression of improvement; QoL, quality of life; IEF, incontinence episode frequency; SUI, stress urinary incontinence; UI, urinary incontinence; UIW, UI in women.

**Table 3:** GRADE assessment for outcomes.

Certainty assessment							No of patients		Effect		Certainty	Importance
**No of studies**	**Study design**	**Risk of bias**	**Inconsistency**	**Indirectness**	**Imprecision**	**Other considerations**	**mobile apps**	**usual care**	**Relative (95% CI)**	**Absolute (95% CI)**		
**Adherence**												
4	Randomized trials	Not serious	Not serious	Not serious	Not serious	None	189/225 (84.0%)	138/233 (59.2%)	RR 1.42 (1.26 to 1.59)	81 more per 1,000 (from 54 more to 106 more)	⊕⊕⊕⊕ High	CRITICAL
ICIQ-UI SF												
11	Randomized trials	Not serious	Very serious^a^	Not serious	Not serious	None	742	745	-	MD 1.93 lower (2.87 lower to 0.98 lower)	⊕⊕○○ Low	CRITICAL
ICIQ-LUTSqol												
4	Randomized trials	Not serious	Very serious^b^	Not serious	Not serious	None	315	317	-	MD 2.78 lower (4.43 lower to 1.14 lower)	⊕⊕○○ Low	CRITICAL
incontinence episode frequency												
5	Randomized trials	Not serious	Very serious^c^	Not serious	Not serious	None	374	377	-	MD 2.28 lower (3.62 lower to 0.94 lower)	⊕⊕○○ Low	CRITICAL
PGI-I												
4	Randomized trials	Not serious	Very serious^d^	Not serious	Not serious	None	237/348 (68.1%)	95/352 (27.0%)	RR 3.79 (1.27 to 11.29)	753 more per 1,000 (from 73 more to 1,000 more)	⊕⊕○○ Low	IMPORTANT
Power												
2	Randomized trials	Not serious	Not serious	Not serious	Not serious	None	93	94	-	MD 0.09 lower (0.24 lower to 0.07 higher)	⊕⊕⊕⊕ High	IMPORTANT
**Endurance**												
2	Randomized trials	Not serious	Not serious	Not serious	Not serious	None	93	94	-	MD 0.08 lower (0.42 lower to 0.26 higher)	⊕⊕⊕⊕ High	IMPORTANT
Repetitions												
2	Randomized trials	Not serious	Serious^e^	Not serious	Not serious	None	93	94	-	MD 0.17 higher (0.57 lower to 0.91 higher)	⊕⊕⊕○ Moderate	IMPORTANT
Fast												
2	Randomized trials	Not serious	Not serious	Not serious	Not serious	None	93	94	-	MD 0.07 higher (0.26 lower to 0.4 higher)	⊕⊕⊕⊕ High	IMPORTANT

CI: confidence interval; MD: mean difference; RR: risk ratio. Explanations.

a. I^∧^2 = 93%.

b. I^∧^2 = 78%.

c. I^∧^2 = 94%.

d. I^∧^2 = 96%.

e. I^∧^2 = 61%.

### 3.3 Meta-analysis

#### 3.3.1 Adherence

**Figure 3:**
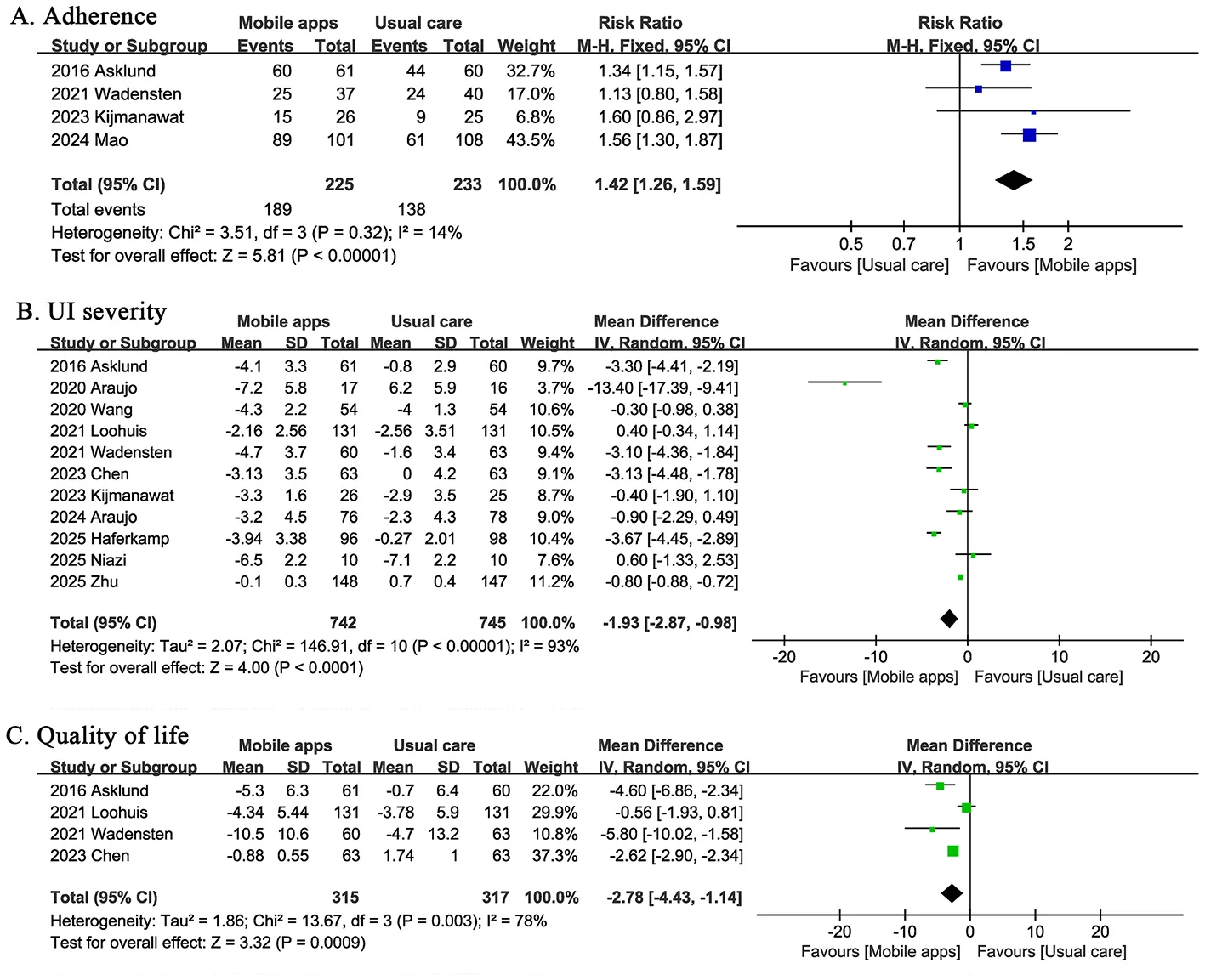
Comparison of primary outcomes between the two groups. **A**. adherence, **B**. ICIQ-UI SF scores, and **C**. ICIQ-LUTSqol scores.

Four studies assessed adherence. The pooled analysis indicated that mobile app–based interventions significantly improved adherence to PFMT (RR 1.42, 95% CI 1.26, 1.59, *P* < 0.0001; Heterogeneity: *I*^2^ = 14%, *P* = 0.32; Figure 3A; Table 3).

#### 3.3.2 UI severity

UI severity was reported in 11 studies. The pooled results of this meta-analysis showed that mobile app-based interventions significantly reduced ICIQ-UI SF scores (MD, −1.93; 95% CI, −2.87, −0.98, *P* < 0.0001; Heterogeneity: *I*^2^ = 93%, *P* < 0.00001; Figure 3B).

#### 3.3.3 Quality of life

Quality of life was reported in 4 studies. The pooled analysis showed that mobile app-based interventions significantly reduced ICIQ-LUTSqol scores (MD, −2.78; 95% CI, −4.43, −1.14, *P* = 0.0009; Heterogeneity: *I*^2^ = 78%, *P* = 0.003; Figure 3C).

#### 3.3.4 PGI-I

**Figure 4:**
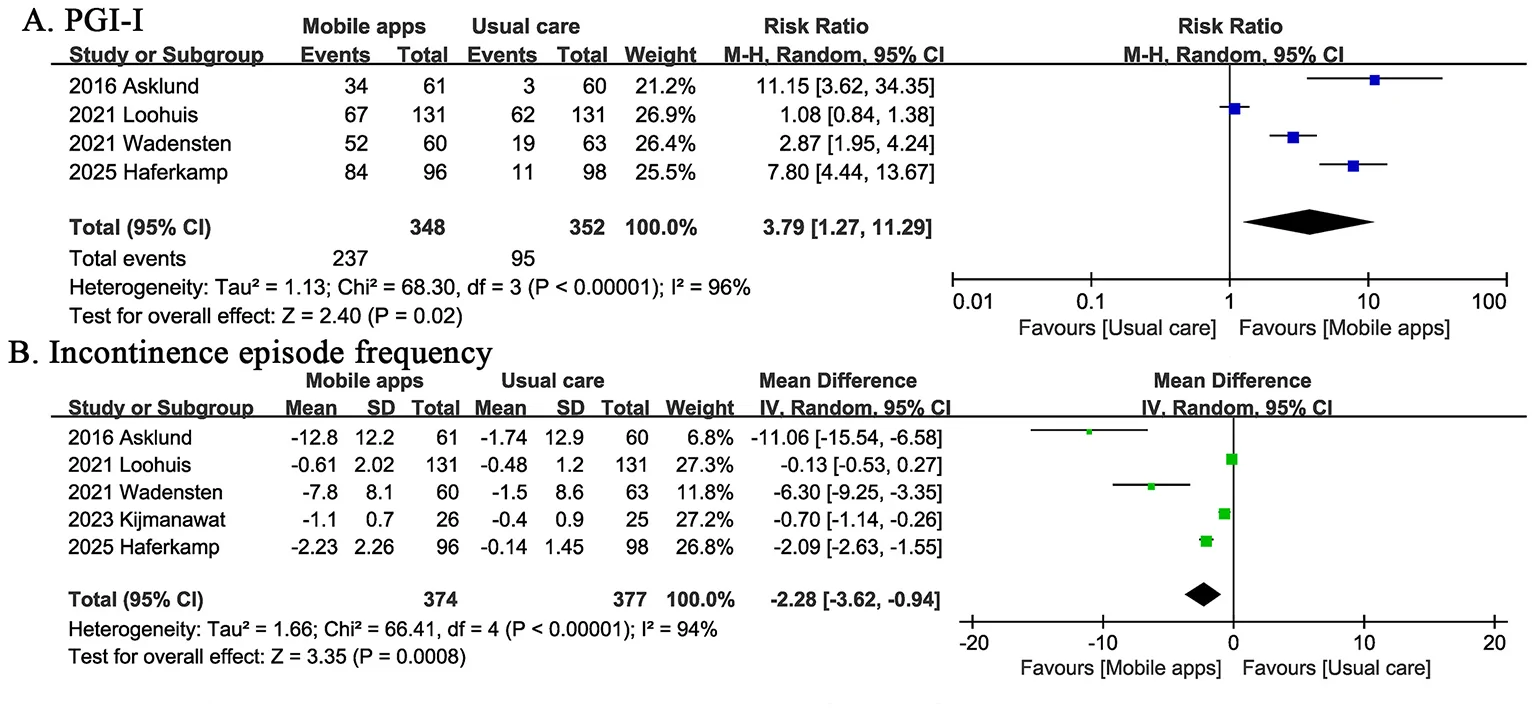
Comparison of secondary outcomes between the two groups. **A**. PGI-I and **B**. incontinence episode frequency.

Four studies provided information on PGI-I. The pooled analysis indicated that mobile app-based interventions significantly improved PGI-I (RR 3.79, 95% CI 1.27, 11.29, *P* = 0.02; Heterogeneity: *I*^2^ = 96%, *P* < 0.00001; Figure 4A).

#### 3.3.5 Incontinence episode frequency

Five trials reported the incontinence episode frequency. The pooled analysis showed that mobile app-based interventions significantly reduced the incontinence episode frequency (MD, −2.28; 95% CI, −3.62, −0.94, *P* = 0.0008; Heterogeneity: *I*^2^ = 94%, *P* < 0.00001; Figure 4B).

#### 3.3.6 Pelvic floor muscle function

**Figure 5:**
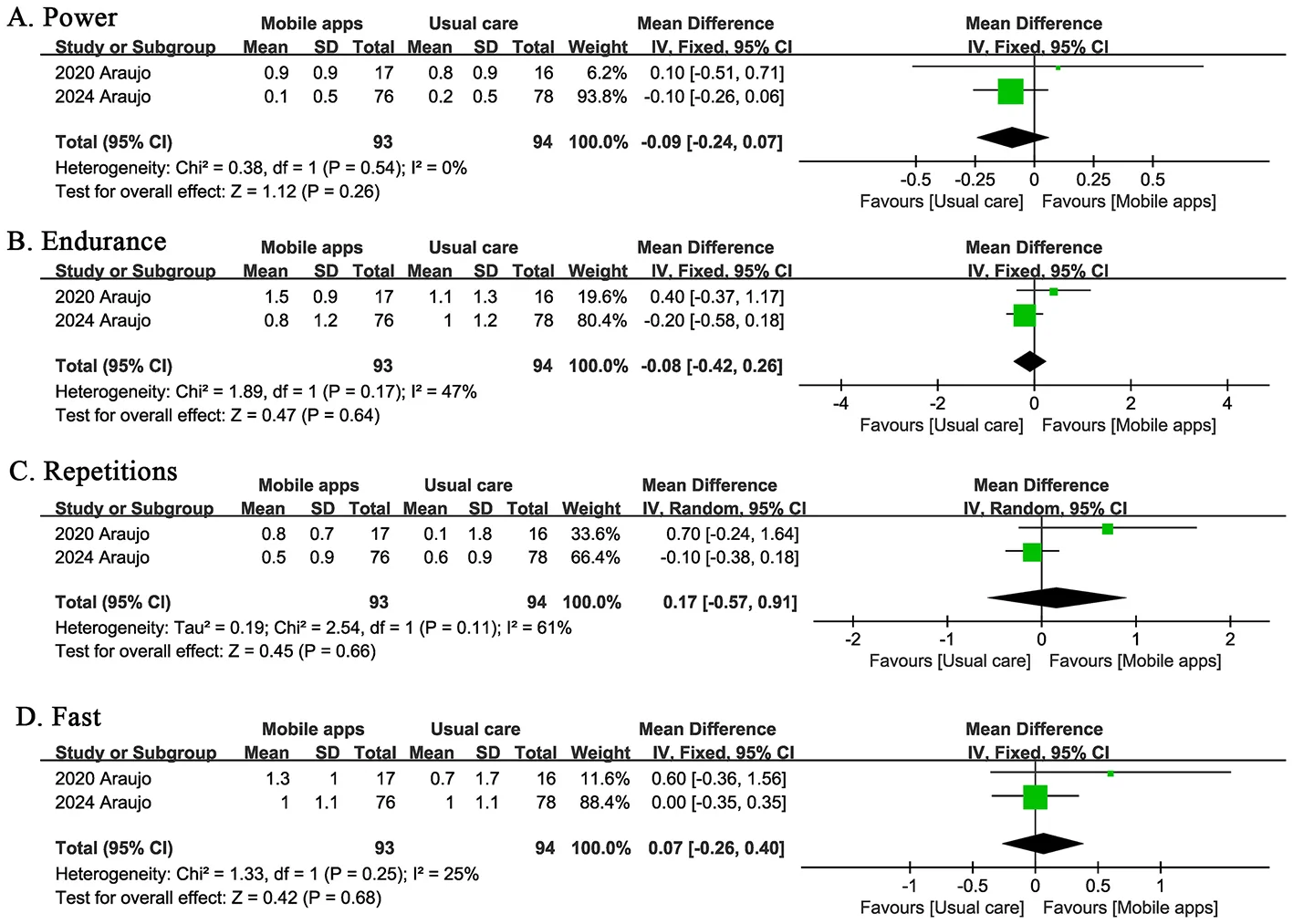
Comparison of secondary outcomes between the two groups in Pelvic floor muscle function. **(A)** power, **(B)** endurance, **(C)** repetitions, and **(D)** fast.

Two studies involving a total of 187 women with UI evaluated the effects of mobile app–based interventions on pelvic floor muscle function. The pooled results indicated that mobile app–based interventions had no significant effect on power (MD −0.09, 95% CI −0.24, 0.07, *P* = 0.26; Heterogeneity: *I*^2^ = 0%, *P* = 0.54), endurance (MD −0.08, 95% CI −0.42, 0.26, *P* = 0.64; Heterogeneity: *I*^2^ = 47%, *P* = 0.17), repetitions (MD 0.17, 95% CI −0.57, 0.91, *P* = 0.66; Heterogeneity: *I*^2^ = 61%, *P* = 0.11), or fast contractions (MD 0.07, 95% CI −0.26, 0.40, *P* = 0.68; Heterogeneity: *I*^2^ = 25%, *P* = 0.25; Figure 5).

### 3.4 Publication bias and sensitivity analysis

**Figure 6:**
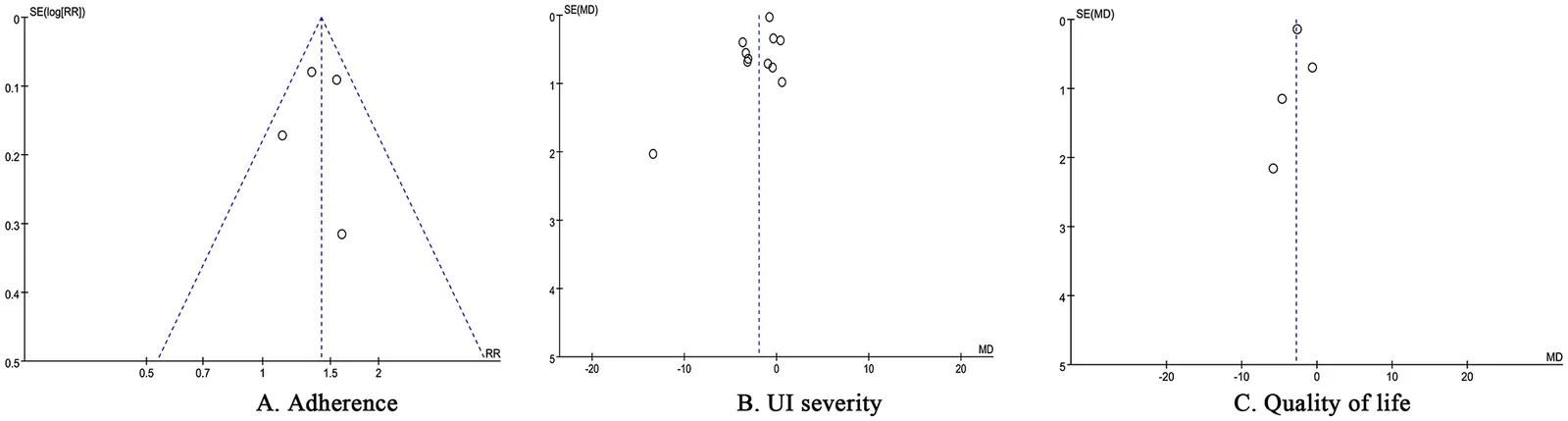
Funnel plot of primary outcomes. **A**. adherence, **B**. ICIQ-UI SF scores, and **C**. ICIQ-LUTSqol scores.

According to funnel plots and Egger's test (Figure 6), no significant publication bias was observed for adherence (*P* = 0.169), ICIQ-UI SF (*P* = 0.136), and ICIQ-LUTSqol (*P* = 0.883). Sensitivity analysis showed that no single study affected the overall effect size of the ICIQ-UI SF, adherence, ICIQ-LUTSqol, incontinence episode frequency, and PGI-I.

### 3.5 Subgroup analysis

Owing to the limited number of studies reporting the other outcomes, subgroup analyses were performed only for UI severity. Subgroup analyses according to intervention duration showed that app-based interventions significantly improved ICIQ-UI SF scores in both the ≥3-month subgroup (MD, −2.41; 95% CI, −4.13 to −0.69; *P* = 0.006; *I*^2^ = 95%) and the < 3-month subgroup (MD, −1.66; 95% CI, −3.21 to −0.11; *P* = 0.04; *I*^2^ = 89%). However, a greater benefit was observed in studies with intervention durations ≥3 months. No significant heterogeneity was detected between the two subgroups (*P* = 0.53, *I*^2^ = 0%). Subgroup analyses according to intervention type further demonstrated significant improvements in ICIQ-UI SF scores in both the simple PFMT app subgroup (MD, −2.78; 95% CI, −5.42 to −0.14; *P* = 0.04; *I*^2^ = 93%) and the multifunctional digital therapeutic system subgroup (MD, −1.88; 95% CI, −3.09 to −0.67; *P* = 0.002; *I*^2^ = 94%). No significant heterogeneity was observed between the two subgroups (*P* = 0.54, *I*^2^ = 0%).

## 4 Discussion

This systematic review aimed to summarize the evidence regarding the effectiveness of mobile app-based PFMT in improving UI severity, adherence, PGI-I, quality of life, and pelvic floor muscle function. Based on 12 RCTs involving 1,647 participants (mobile app group: 823; usual care group: 824), this meta-analysis demonstrated that mobile app-based interventions were associated with improvements in UI severity, quality of life, and frequency of incontinence episodes, while also enhancing patient adherence and PGI-I. However, according to the GRADE framework, the certainty of evidence for several critical outcomes, including ICIQ-UI SF, ICIQ-LUTSqol, incontinence episode frequency, and PGI-I, was rated as low, primarily due to substantial heterogeneity across studies. Therefore, these findings should be interpreted cautiously, and mobile app-based PFMT should currently be considered a supportive strategy to enhance adherence and patient engagement rather than a replacement for supervised rehabilitation.

Symptom relief and improvement in quality of life are the primary goals for both women with UI and healthcare providers (24). In the present review, 11 of the included studies assessed UI symptom severity using the ICIQ-UI SF. Previous studies have suggested that the minimal important difference (MID) for the ICIQ-UI SF is 2.5 points (14, 25). Among the 11 included studies, reductions in ICIQ-UI SF scores exceeded the MID threshold in 9 studies in the app-based intervention group, whereas only 4 studies in the control group achieved reductions greater than 2.5 points. Consistent with these findings, the pooled analysis demonstrated a significantly greater reduction in ICIQ-UI SF scores among women receiving mobile app-based interventions (MD = −1.93). However, the pooled effect did not reach the reported MID threshold. Therefore, although the findings indicate a statistically significant improvement in UI symptom severity, the overall clinical importance of this effect remains uncertain and warrants further investigation in future high-quality studies. Notably, substantial between-study heterogeneity was observed for this outcome. To explore potential sources of heterogeneity, we conducted subgroup analyses according to intervention duration and app functionality. App-based interventions significantly improved ICIQ-UI SF scores in both the ≥3-month and < 3-month subgroups. Although the pooled effect estimate was numerically greater in studies with intervention durations of at least three months, the test for subgroup differences was not statistically significant. Therefore, there is currently no statistical evidence that longer intervention duration is associated with greater treatment effectiveness than shorter intervention duration. Interestingly, subgroup analyses suggested that both simple reminder-based applications and more sophisticated digital therapeutic systems were associated with symptom improvement, with no statistically significant subgroup differences. This finding implies that enhancing adherence may be the principal mechanism underlying the effectiveness of current mobile applications rather than the complexity of digital functions alone. However, digital therapeutic platforms integrating individualized exercise prescription, symptom monitoring, remote clinician feedback, and behavioral coaching may provide additional long-term value that was insufficiently evaluated in the current evidence. These subgroup analyses should be interpreted cautiously because they were exploratory in nature, involved a limited number of studies, and substantial residual heterogeneity remained within both subgroups. Further high-quality studies are warranted to clarify the influence of intervention duration and app functionality on the effectiveness of mobile app-based PFMT.

Regarding quality of life, our meta-analysis demonstrated that app-based interventions significantly improved ICIQ-LUTSqol scores among women with UI. Several previous studies support our findings. Araujo et al. (26) conducted a single-arm clinical study involving 156 women (mean age: 49.3 ± 14.2 years) during the COVID-19 pandemic and reported significant improvements in UI symptoms and quality of life following app-guided PFMT. Notably, 74.3% of participants adhered to the recommended training frequency, and 62% reported complete or substantial symptom improvement. Similarly, Hua et al. (27) demonstrated that a mobile app-based intervention specifically designed for UI in women (UIW) effectively reduced the severity of postpartum UI, potentially through improvements in self-efficacy. Berre et al. (28) reported that a 12-week online PFMT program significantly alleviated UI symptoms at 6-month follow-up. In addition, a meta-analysis by Huang et al. indicated that telemedicine interventions significantly reduced UI severity and improved quality of life compared with usual care. These findings are consistent with the results of the present study (5). Notably, the certainty of evidence for both ICIQ-UI SF and ICIQ-LUTSqol outcomes was rated as low. Therefore, additional high-quality studies are needed to further validate the benefits of app-based interventions for women with UI.

PGI-I is a validated, patient-reported outcome measure that assesses women's overall perception of treatment-related improvement. In the present meta-analysis, women receiving mobile app–based interventions were significantly more likely to report that their symptoms were “much improved” or “very much improved” than those receiving usual care. This finding is consistent with the observed improvements in self-reported symptom severity and quality of life, suggesting that mobile app-based PFMT may enhance women's perceived treatment benefits.

Regarding adherence, our meta-analysis demonstrated that mobile app–based interventions significantly improved adherence to PFMT. This finding is consistent with previous studies, such as those by Liu et al. (9) and Widdison et al. (11), which demonstrated the beneficial role of mHealth in enhancing medication adherence, self-efficacy, patient satisfaction, and symptom control. Several mechanisms may explain the improved adherence observed with mobile apps. First, mobile apps allow women with UI to perform exercises anytime and anywhere, eliminating the need for frequent hospital visits and thereby increasing convenience and engagement. Second, interactive and user-friendly features can enhance motivation and promote sustained participation. Third, mobile apps can function as digital coaches by providing timely reminders and encouraging adherence to prescribed exercise regimens. Clinicians can also design personalized, stage-based training programs and leverage reminder functions to help women with UI establish long-term exercise habits (3). Importantly, adherence is influenced by multiple factors, including individual characteristics, app-related features, and patient awareness. Wessels et al. (29) suggested that these factors may act as either facilitators or barriers depending on user context. Understanding these determinants is essential for optimizing intervention design. Potential strategies for improving mobile apps include: (a) language localization, such as multilingual support and voice-guided instructions tailored to local dialects; (b) optimization for low-resource settings, including offline functionality or low-bandwidth solutions to enhance accessibility in rural areas; and (c) incorporation of culturally relevant motivational elements, such as family involvement in collectivist societies or stigma-reduction features (4).

With respect to pelvic floor muscle function, most of the included studies relied on patient-reported outcome measures, whereas objective assessments were limited. Only two studies evaluated the effects of mobile app–based interventions on pelvic floor muscle function. Both studies used the PERFECT assessment, a standardized digital vaginal palpation method that evaluates Power, Endurance, Repetitions, Fast contractions, and Every Contraction Timed, to assess pelvic floor muscle performance. The absence of measurable improvement in pelvic floor muscle function despite improvements in subjective outcomes deserves particular attention. Several explanations are possible. First, symptom perception is influenced not only by muscle strength but also by behavioral adaptation, bladder training, improved exercise adherence, and increased confidence in symptom self-management, all of which may improve patient-reported outcomes without producing detectable physiological changes. Second, most mobile applications primarily function as behavioral support tools through reminders, education, and exercise guidance rather than providing real-time biofeedback or supervision to ensure correct muscle contraction. Consequently, women may perform PFMT more consistently but not necessarily more effectively. Third, objective muscle assessment was available in only two small trials, limiting statistical power. Consequently, the current evidence is insufficient to determine whether mobile app–based interventions can improve objective pelvic floor muscle function. Further well-designed randomized controlled trials incorporating standardized objective assessments are warranted to clarify the effects of mobile app–based PFMT on pelvic floor muscle function.

This study has several strengths. First, a comprehensive literature search was conducted across five major databases (PubMed, Cochrane Library, Scopus, EMBASE, and Web of Science). Second, only randomized controlled trials were included, thereby minimizing potential bias and enhancing the reliability of the findings.

However, several limitations should be acknowledged. First, some included studies had relatively small sample sizes, which may affect the stability of the results. Second, substantial heterogeneity was observed in certain outcomes. This heterogeneity may be attributable to differences in app functionality, intervention duration and follow-up schedules, healthcare systems and cultural contexts, and study eligibility criteria across the included trials. Although subgroup analyses were performed where possible, considerable residual heterogeneity remained. In addition, considerable variation also exists in the populations included across studies. Younger postpartum women often develop UI secondary to pregnancy-related pelvic floor injury and generally have good rehabilitation potential. In contrast, postmenopausal women frequently experience chronic pelvic floor dysfunction associated with aging, estrogen deficiency, and multiple comorbidities. These differences in pathophysiology and rehabilitation needs may contribute to heterogeneity across studies, particularly between younger postpartum women and older postmenopausal women, whose treatment preferences and expectations for UI management may differ substantially. Future trials should stratify participants according to age and clinical phenotype to facilitate more individualized digital interventions. Third, relatively few studies were available for several outcomes, including adherence, pelvic floor muscle function, and quality of life assessed by the ICIQ-LUTSqol, thereby limiting the robustness of the pooled estimates for these outcomes. In particular, objective assessments of pelvic floor muscle function were available in only two studies, whereas most included studies relied primarily on patient-reported outcome measures. Fourth, the applicability of mobile app–based interventions in rural or older adults requires further investigation. Furthermore, publication bias was assessed using funnel plots and Egger's test; however, Egger's test may be unreliable when the number of included studies is limited. In addition, most of the included studies had relatively short follow-up periods, making it difficult to determine whether the observed benefits of mobile app-based interventions can be sustained over the long term. Finally, although mobile apps show considerable promise in women with UI, their effectiveness in male populations remains to be established.

In conclusion, this meta-analysis suggests that Mobile app–based PFMT appears to be a promising adjunctive strategy for women with UI by improving adherence, symptom severity, quality of life, and patient-reported improvement. However, the pooled reduction in symptom severity did not reach the established minimal important difference, objective pelvic floor muscle function was not significantly improved, and the certainty of evidence for several key outcomes was low. Therefore, current evidence supports the integration of mobile applications as adjuncts rather than replacements for supervised PFMT. Future high-quality trials incorporating objective functional assessment, longer follow-up, and digital therapeutic platforms are warranted.

## Data Availability

The original contributions presented in the study are included in the article/supplementary material, further inquiries can be directed to the corresponding author.
